# Biophotonic detection of high order chromatin alterations in field carcinogenesis predicts risk of future hepatocellular carcinoma: A pilot study

**DOI:** 10.1371/journal.pone.0197427

**Published:** 2018-05-17

**Authors:** Richard Kalman, Andrew Stawarz, David Nunes, Di Zhang, Mart A. Dela Cruz, Arpan Mohanty, Hariharan Subramanian, Vadim Backman, Hemant K. Roy

**Affiliations:** 1 Department of Medicine, Einstein Medical Center, Philadelphia, Pennsylvania, United States of America; 2 Biomedical Engineering Department, Northwestern University, Evanston, Illinois, United States of America; 3 Department of Medicine, Boston University Medical Center, Boston, Massachusetts, United States of America; University of Colorado, UNITED STATES

## Abstract

**Purpose:**

Hepatocellular carcinoma (HCC) results from chronic inflammation/cirrhosis. Unfortunately, despite use of radiological/serological screening techniques, HCC ranks as a leading cause of cancer deaths. Our group has used alterations in high order chromatin as a marker for field carcinogenesis and hence risk for a variety of cancers (including colon, lung, prostate, ovarian, esophageal). In this study we wanted to address whether these chromatin alterations occur in HCC and if it could be used for risk stratification.

**Experimental design:**

A case control study was performed in patients with cirrhosis who went on to develop HCC and patients with cirrhosis who did not develop cancer. We performed partial wave spectroscopic microscopy (PWS) which measures nanoscale alterations on formalin fixed deparaffinized liver biopsy specimens, 17 progressors and 26 non-progressors. Follow up was 2089 and 2892 days, respectively.

**Results:**

PWS parameter disorder strength *L*_*d*_ were notably higher for the progressors (*L*_*d*_ = 1.47 ± 0.76) than the non-progressors (*L*_*d*_ = 1.00 ± 0.27) (p = 0.024). Overall, the Cohen’s d effect size was 0.907 (90.7%). AUROC analysis yielded an area of 0.70. There was no evidence of confounding by gender, age, BMI, smoking status and race.

**Conclusions:**

High order chromatin alterations, as detected by PWS, is altered in pre-malignant hepatocytes with cirrhosis and may predict future risk of HCC.

## Introduction

Hepatocellular carcinoma (HCC) is the 2^nd^ most common cause of cancer related mortality in men and 6^th^ most common cause in women world-wide [1], and the incidence is rapidly increasing in Western countries which emphasizes the need for effective cancer preventive strategies [2,3]. The prognosis for advanced HCC remains poor, underscoring the need for early detection efforts [4]. These efforts are aided by the established risk factor for HCC being cirrhosis (responsible for ~90% of cases). Screening is advocated for cirrhotics with ultrasound with or without assessment of serum alpha fetoprotein (AFP). However, given the low prevalence of HCC (progression from cirrhosis to HCC estimated to be ~2–4% per year) and the non-specificity of screening tests, screening for HCC is plagued not only by poor sensitivity but also large numbers of false positives [4]. Other issues include impact of gender, race and etiology making personalizing screening strategies of high importance. HCC has at least three distinct biological patterns with methylation, microRNA and copy number alterations that add to the complexity of this malignancy [5]. Therefore, in order to personalize screening strategies, it is critical to understand early events in hepatic carcinogenesis.

Our group has focused on nano-architectural changes in early carcinogenesis [6]. Using a novel biophotonics technique, partial wave spectroscopic microscopy (PWS), we have found that alterations in chromatin nano-architecture is one of the earliest events in neoplastic transformation. Indeed, this occurs during field carcinogenesis, the notion that the field of injury (genetic/exogenous factors) is noted in a variety of cancer types. Etiological field carcinogenesis has been noted for a variety of tumors including colon, lung, and ovarian [7]. We have demonstrated that PWS related high order chromatin abnormalities can be used to predict risk of carcinogenesis in lung, colon, esophagus, ovarian and prostate cancer [8]. Biologically, we have recently reported that PWS findings correlate closely with gene expression supporting the biological relevance [9,10]. Since the issue of field carcinogenesis in HCC is relatively underexplored, we performed a case-control study in patients with cirrhosis using development of future HCC as our outcome.

## Materials and methods

### Patients

This study was approved by the International Review Board at the Boston Medical Center. As all data was collected retrospectively, requirement for consent was waived. No minors were included in this study. Patients included in this case control study were cirrhotics that were divided into two groups: 1) those who later developed HCC (termed progressors) and 2) those who remained free of HCC (termed non-progressors). Inclusion criteria consisted of having a liver biopsy from 2000–2012 for routine clinical care. Progressors required a confirmed diagnosis of HCC upon follow-up after an initial negative biopsy was obtained. HCC was diagnosed by either biopsy or standard radiologic criteria. HCC diagnosed by histopathology typically have a polygonal shape with granular and eosinophilic cytoplasm in a trabecular pattern; tumor cells may secrete bile which is a unique feature of HCC. Non-progressors required a liver biopsy confirming cirrhosis and imaging negative for HCC for at least 5 years after biopsy. For this study, a total of 26 non-progressor and 17 progressor biopsy specimens were successfully obtained (total n = 43). Additional patient information included demographic factors and clinical measures of fibrosis/cirrhosis (i.e. AFP, AST, ALT, F score, HAI score, etc.). Progressors were determined to fall within or outside the standard Milan criteria for liver transplant based on initial tumor presentation. The Milan criteria was established to detetrmine which patients with HCC can safely move on to liver transplant based on relatively small risk for recurrent HCC and requires that the cancer at diagnosis include either a single lesion less than five cm or up to three lesions each less than three cm [3].

### Samples

Tissue samples were obtained from the cirrhosis patients undergoing routine liver biopsy (via ultrasound-guided percutaneous biopsy or transjugular biopsy). Samples were then formaldehyde-fixed and paraffin-embedded, sectioned into 5 μm slices, and prepared on glass slides with and without staining (H&E). Histology was studied using the H&E stained slides to verify the presence of hepatocytes while acquired measurements with the PWS microscope were done on unstained slides after deparaffinization.

### Partial wave spectroscopic microscopy

PWS is a novel technology whose theory has been described elsewhere [11]. Ultimately, it is a combination of microscopy and spectroscopy whereby an incoherent white light source is shone on a biological sample, and the spectrally-resolved microscope image collected is registered within the visible wavelength range (500–700 nm with 1 nm spacing). The image captured from a charge-coupled device contains nanoscale-sensitive spectral information from collected light that is backscattered as a result of refractive index mismatch (illumination NA = 0.1 and collection NA = 0.4). The amplitude of spectral oscillations resulting from interference between waves scattered from within the sample and a reference wave reflected from the air-sample interface results in the PWS signal. Because refractive index is directly proportional to mass density within certain organelles, PWS is sensitive to the spatial distribution of mass density [12,13]. It has been previously shown that PWS is sensitive to length scales within the size range of 20–200 nm; less than the diffraction limit of light used in conventional microscopy [14]. The quantity used to statistically measure the spatial distribution of mass density within the cells that provides an indication of nanoscale architecture is called disorder strength (*L*_*d*_). In essence, this quantity is a measure of the inhomogeneity of nanoscale structure. For small length scales (on the order of tens of nanometers), the following equation is accurate to quantify disorder strength:

$$L_{d}=\sigma_{n}lc$$

Where *σ*_*n*_ is standard deviation and *lc* is correlation length of the spectral fluctuations of refractive index [15].

### Data acquisition and analysis

Measurements of *L*_*d*_ were taken from unstained slides within 3 days after deparaffinization. A total of 15 images were obtained with the PWS microscope with two regions of interests per image (total of 30 regions of interest per sample). Regions of interest were taken from the entire half-regions of the field of view for each image (~95 μm in diameter) being careful to avoid lumen (from steatosis among other causes). Based on stained histology, hepatocytes are roughly 20–30 microns in diameter (using red blood cells to determine length scale which are known to be 6–7 microns in diameter). Therefore, each region of interest measures approximately 10–20 hepatocytes. All measurements were taken with reference to those from a fully reflective mirror. All samples were shipped in 5 batches, and non-progressors were normalized to progressors to account for sample and instrumentation variabilities. In order to eliminate light saturation, images were processed to exclude overexposing pixels. Finally, a low order polynomial subtraction was applied to remove spectral slopes originating from sample roughness as described previously [16].

### Statistical analysis

In order to test for significance of PWS measurements, a two-tailed t-test was performed between progressors and non-progressors for differences in normalized *L*_*d*_ (p<0.05 considered significant). A similar test and a chi-squared test was performed to determine if progressors and non-progressors were different based on continuous demographic factors (i.e. age, fibrosis stage and BMI) and categorical demographic factors (i.e. gender, smoking, race, etc), respectively. Effect size was calculated using Cohen’s d, which is the difference between means over the pooled standard deviation (effect size of >0.80 or 80% considered large). To determine if these demographic factors were confounding factors, analysis of covariance analysis (ANCOVA) was performed with normalized *L*_*d*_ as the dependent variable, progressors and non-progressors as the independent variable, and one of the demographic factors as the covariate. Because of the assumption of homogeneity of regression slopes, gender was not appropriate for ANCOVA since its interaction with the independent variable was significant (thus breaking an underlying assumption of ANCOVA). Alternatively, a two-tailed t-test was performed for normalized *L*_*d*_ between the groups within categorical demographic factors (gender, smoking, and race). A receiver operating characteristic (ROC) curve was also evaluated to determine the performance (area under ROC (AUROC), sensitivity, and specificity) of PWS to differentiate between progressors and non-progressors. T-test analysis, chi-squared analysis and effect size was performed using Microsoft Excel and ANCOVA and system performance was performed using MATLAB R2013a.

## Results

A total of 1,214 liver biopsy specimens were reviewed at Boston Medical Center from 2000–2012. A total of 54 patients met criteria as progressors and 17 histologic samples were retrieved from pathology. In the non-progressor category, 75 patients were identified that met criteria and 26 specimens were successfully retrieved for analysis.

The average age of progressors was 51.3 years, 82% were male and the majority had Hepatitis C virus (HCV) (Table 1). The non-progressors mostly had liver disease attributed to HCV as well, and the average age was 50.4. The majority of non-progressors were also male (62%). Distribution of race and BMI were similar between the two groups. In the progressor group, the mean number of days between biopsy and HCC diagnosis was 2,089 days (Table 2). Twelve of 17 patients met Milan criteria at the time of diagnosis, seven patients underwent curative treatment and six were documented as still being alive at the time of the statistical analysis. The non-progressor group had a mean of 2,892 days between index biopsy and most recent negative imaging study (Table 2).

**Table 1:** Baseline demographics of patients in the progressor and non-progressor group.

	Progressors (n = 16)	Non-progressors (n = 20)	P value
Age, y, mean (SD)	51.6 (+/-8.5)	51.4 (+/-6.8)	0.93
Male gender (%)	13 (81%)	11 (55%)	0.10
Race (%)			0.72
Asian	1 (6%)	1/20 (5%)	
African American	6 (38%)	7/20 (35%)	
Caucasian	6 (38%)	5/20 (25%)	
Hispanic or Latino	3 (18%)	7/20 (35%)	
BMI, mean (SD)	29.3 (+/-3.4)	29.7 (+/-7.3)	0.82
History of smoking	12	15	1.0
Etiology			0.52
HCV	10	10	
HCV + EtOH	3	2	
HCV + HIV	0	3	
HCV + HBV	0	1	
HCV + NASH	0	1	
HCV + HIV + EtOH	1	1	
HBV	2	1	
NASH	0	1	
Fibrosis stage, mean (SD)	4.4 (+/-1.5)	5.1 (+/- 0.7)	0.11
HAI score, mean (SD)	6.4 (+/-2.3)	7.6 (+/-1.4)	0.08
Albumin, g/dl, mean (SD)	4.0 (+/-0.5)	4.1 (+/-0.4)	0.52
INR, mean (SD)	1.09 (+/-0.11)	1.08 (+/-0.09)	0.66
Bilirubin, mg/dl, mean (SD)	0.79 (+/-0.39)	0.75 (+/-0.71)	0.83
ALT, U/l, mean (SD)	105 (+/-58)	93 (+/-62)	0.55
AST, U/l, mean (SD)	90 (+/-43)	80 (+/-43)	0.49
Platelets, K/Ul mean (SD)	144 (+/-52)	170 (+/-63)	0.18
MELD, mean (SD)	7.9 (+/-1.5)	8.4 (+/-4.8)	0.65
Child Pugh, mean (SD)	5.3 (+/-0.6)	5.2 (+/-0.5)	0.79
Varices	4/16	2/20	0.23

**Table 2:** Progressor details regarding cancer treatment and outcome, and non-progressor details on severity of liver disease.

	Progressors (n = 16)
Time elapsed from biopsy to HCC, mean (range)	2,006 days (504–3741)
Milan criteria	12/16
Curative treatment	6
Outcome	
Died	5
Alive	5
Lost to follow up	6
	Non-Progressors (n = 20)
Time elapsed from biopsy to most recent surveillance, mean (range)	2,790 days (1795–4036)
MELD, mean (SD)	9.3 (+/-3.9)
Child Pugh, mean (SD)	6.6 (+/-2.1)
Varices	6/20

**Fig 1:**
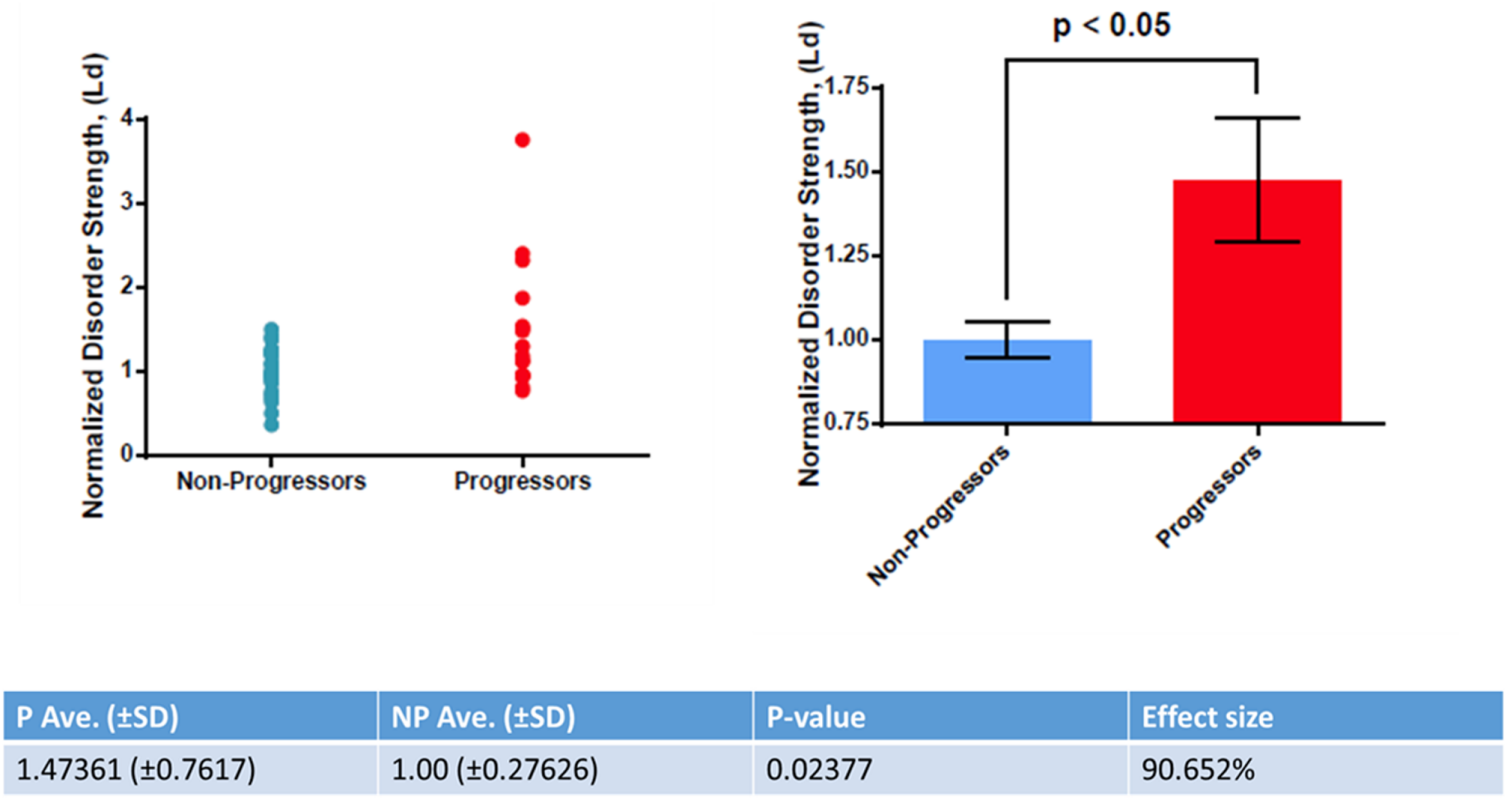
Scatter plot and bar graph illustrating the difference in disorder strength between the non-progressor group and the progressor group.

**Fig 2:**
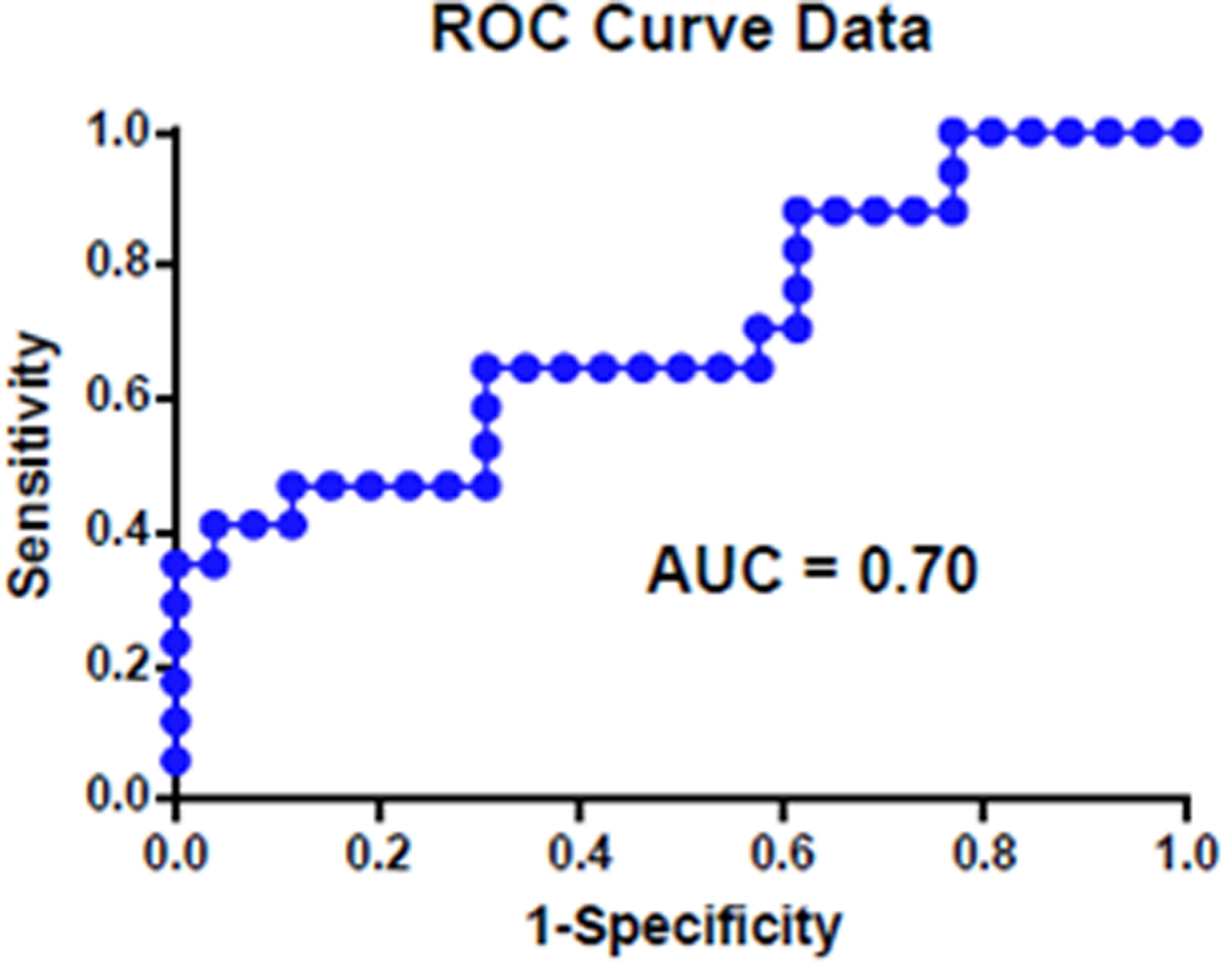
ROC curve illustrating the area under the curve for disorder strength and cancer prediction.

PWS measurement of *L*_*d*_ were notably higher for the progressors (*L*_*d*_ = 1.47 ± 0.76) than the non-progressors (*L*_*d*_ = 1.00 ± 0.27) (p = 0.024). Overall, the Cohen’s d effect size was 0.907 (90.7%) (Fig 1). AUROC analysis yielded an area of 0.70 (Fig 2).

In the progressor group 14 of 17 patients were male and in the non-progressor group 16 of 26 patients were male. Male gender is a known risk factor for HCC, with male to female ratios ranging between 2:1 and over 4:1 across all etiologies of liver disease [17]. The Cohen’s d effect size between progressors and nonprogressors for males was 1.005 (100.5%) and for females 1.758 (175.8%). There was no statistical difference between males and females (t test p value = 0.13). Additionally, there was no significant difference with smoking and race (p = 0.81 and p = 0.23) when comparing progressors and non-progressors.

To assess for confounders, we performed ANCOVA with disorder strength as a dependent variable and predictors including gender, age, BMI, smoking status and race. No significant difference was noted for gender (p 0.28), age (p 0.81), BMI (p 0.42), smoking (p 0.14) and race (p 0.59 when comparing Caucasians to other).

## Discussion

We demonstrate, for the first time, that changes in high order chromatin is one of the earliest changes in liver carcinogenesis. We based this on detection of field carcinogenesis as indicated by development of future HCC. Our proof-of-principle case-control study indicates that PWS measured as *L*_*d*_ was approximately 50% higher in cirrhotics who later developed HCC than those who did not. This effect was maintained when controlled for other risk factors including gender, age, BMI, smoking status and race.

Biologically, one of the challenges of HCC has been its heterogeneity which results in part from the different etiologies of liver injury that might lead to HCC. For instance, hepatitis B virus integrates into the hepatocyte genome, hemochromatosis and non-alcoholic fatty liver (NAFLD) induce oxidative stress while alcohol and HCV are key inflammatory stimuli. Unsupervised clustering analysis of gene expression, methylation, microRNA, DNA copy number indicated that there were three distinct HCC subtypes. Highlighting the complexity of HCC is the observation that subtypes are associated with demographic characteristics. For instance, one with the worst prognosis was associated with female gender, younger age, Asian ethnicity and normal weight. Importantly, regardless of cluster, mutations in chromatin modulators are one of the most common pathways seen in HCC consistent with the emerging role of chromatin modulators in carcinogenesis [10]. Specifically, Arid1a/Arid 2a is mutated in approximately 25% of cancers regardless of HCCs regardless of etiology. Arid 1a is a prototypical SWI-SNF family member important in changing the chromatin architecture by moving nucleosomes to foster gene expression [10,18].

PWS is a remarkable new technology that allows assessing high order chromatin. As a simplified overview it segments the cell into approximately 30,000–50,000 pixels and measures pixel-to-pixel fluctuations in refractive index. Thus, PWS is sensitive to length scales from 20–200 nm. In the nucleus, this allows insight into chromatin packing. Indeed, there are 2 meters of chromatin that needs to be packed into the nucleus [9]. For transcription factors to have access to promotors/enhancers, unravelling the chromatin is critical. This underscores the importance of determination of cancer. The ability of PWS to detect transcriptional activity was highlighted by a recent cell culture study which showed that the Δ *L*_*d*_ related to stimuli such as phorbol esters of EGF correlated with the expected increase in the fraction of overexpressed genes (R^2^ = 0.63) and decrease in the fraction of underexpressed genes (R^2^ = 0.75) independent of the treatment comparison [9,19].

Field carcinogenesis in the liver is relatively underappreciated. Clinically, this is noted given the frequent occurrence of multifocal disease (for instance, the fact that multiple tumors are often encountered at diagnosis and actually included in the standard criteria for liver transplantation with HCC). The biological power of field carcinogenesis was demonstrated by a landmark study by Hoshida and colleagues where using gene expression analysis of paraffin sections, a 186 gene signature was shown to be predictive of survival in HCC patients from normal hepatocytes [20]. This was finding was particularly powerful given that no survival signature was discernable from the actual tumor, presumably related, at least partially, to tumor heterogeneity. MicroRNAs have also been shown to be markers of field carcinogenesis [21]. In addition, Castven and colleagues noted alterations in stem cell markers in field carcinogenesis consonant with the notion that stem cell division was associated with increased risk of carcinogenesis [22]. The complexity of etiology of liver disease, host factors and biological heterogeneity of HCC support the notion that field carcinogenesis is not only the earliest changes but may provide a robust clinical target [21].

While this is predominantly a biological plausibility study, there are potential clinical implications. They include potential for risk stratification especially from staging/diagnostic biopsies. Even though routine biopsies are becoming less common in the era of transient elastography and accurate serologic markers of fibrosis, PWS could still serve as a useful adjunct for determining surveillance protocols for the individual patient if histology is available. Additionally, emerging at risk populations such as those with non-alcoholic steatohepatitis (NASH) and those who have undergone curative HCV therapy may benefit most from HCC risk stratification. HCC has been shown to occur in patients with NASH in the absence of cirrhosis and after successful HCV therapy which makes surveillance strategies more difficult. Many biomarkers of HCC have been discovered in resection specimens limiting their applicability in risk stratification and detection of early stage cancers. As PWS is effective even on biopsies collected more than a decade ago, archived specimens (like those collected prospectively for clinical trials) can be used for development of a biomarker. Patients who underwent biopsies in the past may still benefit from this technology even if subsequent biopsies are not planned.

There are several important strengths. Biologically, this is the first demonstration that chromatin nano-architecture is altered in premalignant hepatocytes. Our recent demonstration that PWS detected chromatin nano-architecture correlates with gene expression changes [9] is particularly apropos to the seminal gene expression-hepatocellular field carcinogenesis work [20]. From a technology perspective, PWS has the potential of being a revolutionary and clinically feasible approach to chromatin nano-architecture. The performance on formalin fixed paraffin embedded tissue enhances utility and this is the first study on nano-architecture for field carcinogenesis in HCC. The follow up was large (mean 2,892 days) and provides rigor for clinical outcomes. Biologically high order chromatin may be a more robust modality of assessing global transcriptional assessment than typical gene arrays. Clinically, HCC is a malignancy with high and rising global burden with suboptimal current screening approaches.

On the other hand, there is a number of limitations that should be acknowledged. The unblinded, retrospective nature of the study limits conclusions about the diagnostic implications of the study. We noted that women had a better diagnostic performance than men. While gender differences have been noted in HCC field carcinogenesis using biomarkers such as microRNA [21], male gender is known to be a risk factor. Finally, as subjects predominantly had viral hepatitis (HCV), better treatment options may decrease this as an etiology of cirrhosis. In this vein, we did not have study subjects with NASH. This is particularly apropos given that while liver biopsies are being eschewed for viral hepatitis, it is still the standard of care for NASH given the limitations of non-invasive technologies (transient elastography or serologic assays).

In conclusion, this pilot study suggests that PWS may serve as a useful method to further risk stratify patients at risk for HCC. Although surveillance for HCC has been shown to improve survival, the majority of patients will derive no benefit from screening and the current modalities including AFP and ultrasound are suboptimal. This study provides rationale for why PWS may be able to help identify low risk patients who do not require surveillance, as well as high risk patients who might benefit from enhanced surveillance, perhaps with MRI instead of ultrasound or more frequent surveillance. Although the AUROC for PWS in this pilot study of patients with cirrhosis at risk for HCC was only 0.70, we believe that with further studies this can be dramatically improved.

## Supporting information

S1 Dataset (XLSX)
