# Correction: Biophotonic detection of high order chromatin alterations in field carcinogenesis predicts risk of future hepatocellular carcinoma: A pilot study

**DOI:** 10.1371/journal.pone.0201500

**Published:** 2018-07-24

**Authors:** Richard S. Kalman, Andrew Stawarz, David Nunes, Di Zhang, Mart A. Dela Cruz, Arpan Mohanty, Hariharan Subramanian, Vadim Backman, Hemant K. Roy

The first author’s middle initial is missing. The correct name is: Richard S. Kalman. The correct citation is: Kalman RS, Stawarz A, Nunes D, Zhang D, Dela Cruz MA, Mohanty A, et al. (2018) Biophotonic detection of high order chromatin alterations in field carcinogenesis predicts risk of future hepatocellular carcinoma: A pilot study. PLoS ONE 13(5): e0197427. https://doi.org/10.1371/journal.pone.0197427
